# Generalized pustular psoriasis in a toddler with IL36RN mutation: a case report

**DOI:** 10.3389/fimmu.2024.1337799

**Published:** 2024-03-20

**Authors:** Ghaith Adi, Mohammed Rami Shaath, Kareem Adi, Zaki Obaid, Egab Aldosari, Faten Ahmed AlKateb

**Affiliations:** ^1^ College of Medicine, Alfaisal University, Riyadh, Saudi Arabia; ^2^ Children’s Specialised Hospital, King Fahad Medical City, Riyadh, Saudi Arabia

**Keywords:** childhood GPP, IL36N gene, gene mutation, psoriasis, generalized pustular psoriasis, biological agents

## Abstract

Generalized Pustular Psoriasis (GPP) is a dermatological autoinflammatory disease that rarely occurs in children and is associated with complex genetic factors. GPP pathogenesis has been associated with mutations in IL36RN gene, which encodes an interleukin-36 receptor antagonist. GPP usually occurs without a history of psoriasis in the patients or their family members. This case report describes the clinical course of a 3-year-old toddler with GPP. The diagnosis of GPP was confirmed through a comprehensive series of examinations, and genetic testing revealed an IL36RN mutation, providing further insight into the genetic basis of the condition. This case highlights the importance of a genetic perspective for diagnosing GPP, particularly in children.

## 1 Introduction

Generalized Pustular Psoriasis (GPP) is a rare and severe form of psoriasis that poses life-threatening risks, characterized by sudden, repeated episodes of diffuse erythema and subcorneal sterile pustules (1). The pustules are primarily infiltrated by sterile neutrophils, which can localize or diffuse throughout the body (2, 3). Individuals with a history of psoriasis vulgaris (PV) commonly suffer from GPP, although it can also occur in those who have never experienced PV (4). Although psoriasis is a chronic immune-mediated skin disorder affecting adults, it is exceedingly rare in the pediatric population, particularly in toddlers (5). The rarity of this case is significant because of associated diagnostic and therapeutic challenges.

## 2 Case presentation

A 3-year-old Saudi male toddler had been suffering from persistent oral ulcers, pruritic punctate papules, and pruritic pustules all over the body for 1 month before his admission to our hospital, that was approximately five months ago. It started with small white blisters on the feet and lower limbs, followed by blisters and erythema on the trunk, head, and mouth. He also presented with a fever, asthenia, and rigor. The patient’s brother has been reported to have similar symptoms simultaneously. The initial differential diagnoses at the local hospital included: Staphylococcal Scalded Skin Syndrome, Bullous Impetigo, Steven Johnson Syndrome, and Toxic epidermal necrolysis (TEN). He was started there on broad-spectrum antibiotics with antiviral therapy to cover a possible herpes simplex virus (HSV) infection because of the severe symptoms; however, he showed no improvement. The patient’s condition partially improved with the empirical administration of fluconazole and betamethasone in the local hospital, and he was referred to a tertiary care hospital (King Fahad Medical City (KFMC)) for further work-up and management. A multidisciplinary team consisting of a histopathologist, dermatologist, and pediatrician collaborates to thoroughly evaluate and manage the patient’s condition.

**Figure 1:**
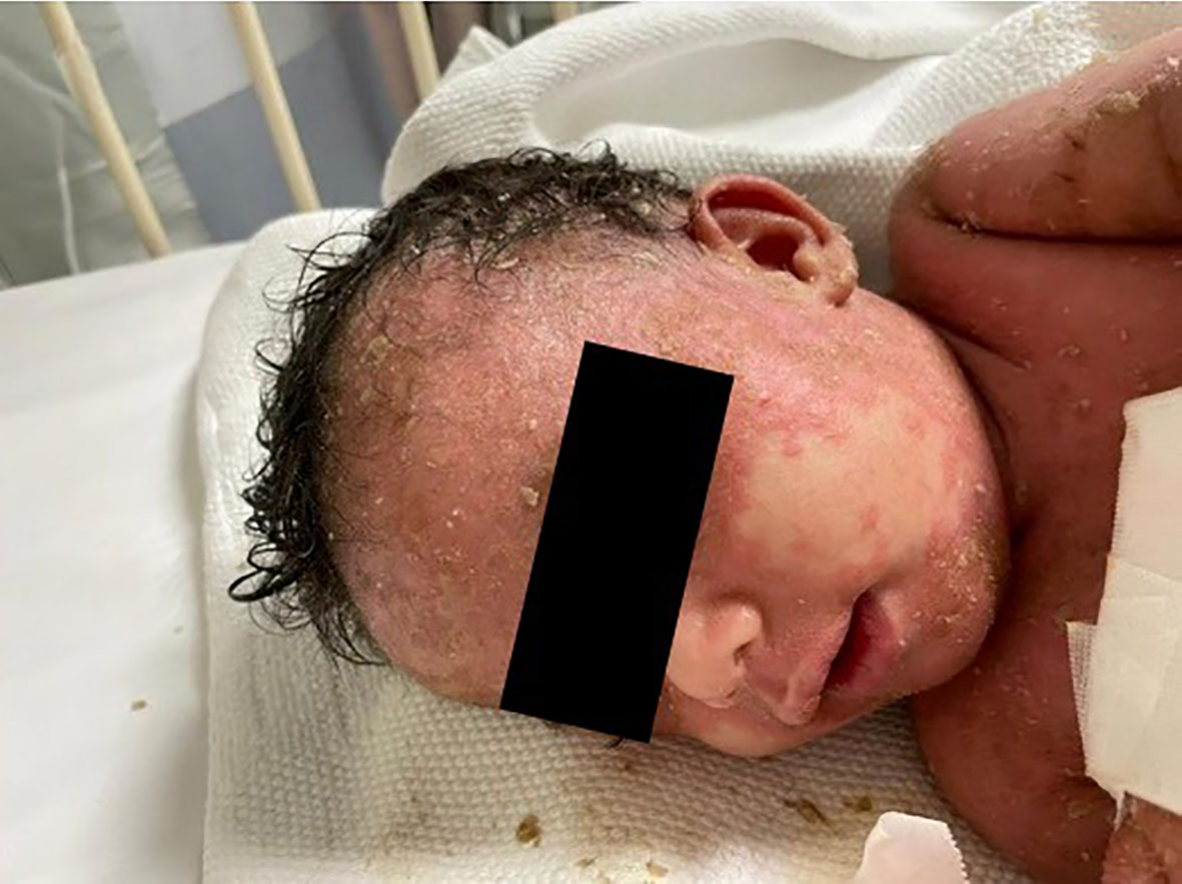
Generalized scaly red rash with desquamation along with small white blisters distributed over the face with pustular lesions.

**Figure 2:**
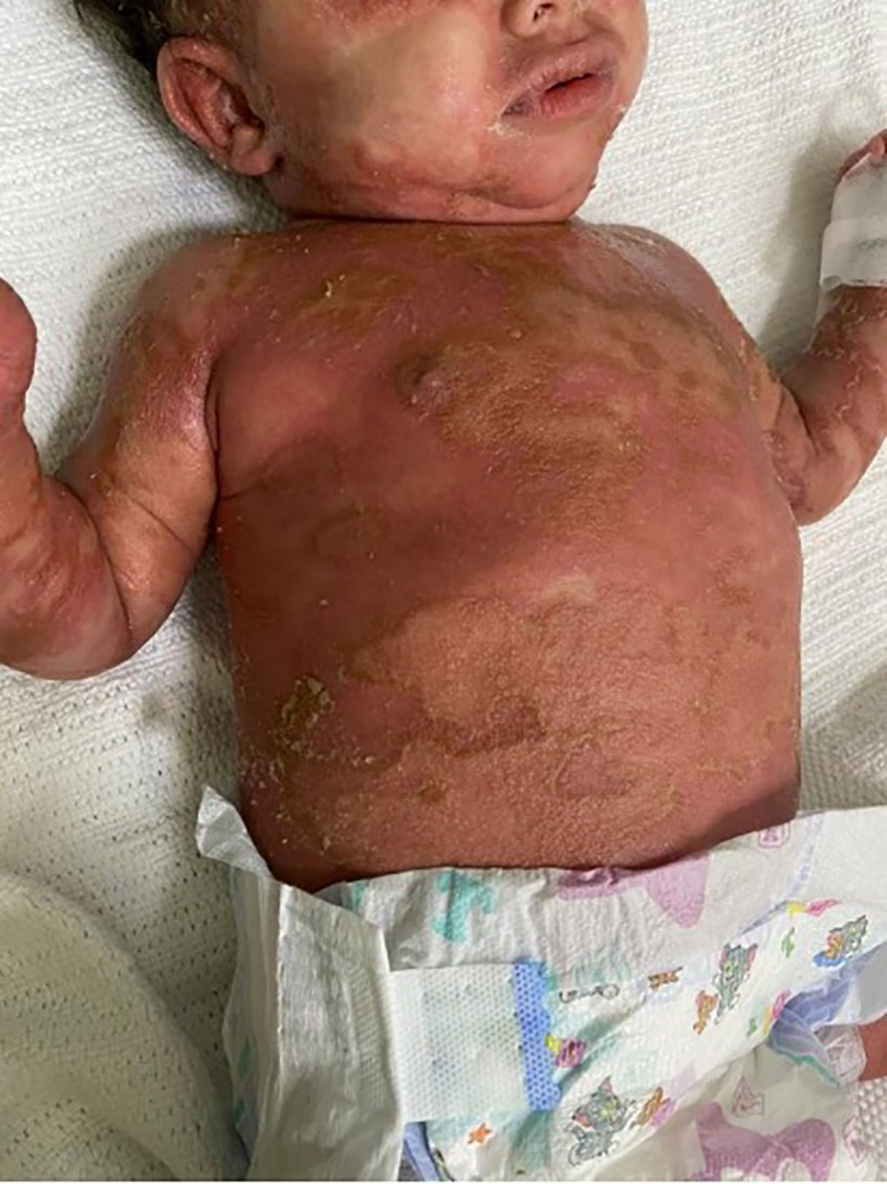
Generalized scaly red rash with desquamation along with small white blisters distributed over the body with pustular lesions.

Physical examination revealed that the patient was unwell, with high-grade persistent pyrexia associated with tachycardia, although he was active and oriented. A full skin examination revealed that the skin was warm, with a scaly rash on the face and abdomen associated with blisters, widespread erythematous plaques, and pustular lesions dispersed across the child’s trunk, extremities, and flexural areas (**Figures 1**, **2**). Furthermore, there was no enlargement of the liver or spleen in the abdomen, and no palpable lymph nodes were found in the cervical, supraclavicular, infraclavicular, axillary, or inguinal regions. These findings indicate the absence of hepatomegaly, splenomegaly, and lymphadenopathy.

Routine laboratory investigations include: CRP levels were significantly elevated at 33.1 (normal: 0.02 to 14.4 mg/l). Additionally, ESR levels were high (107) (normal: 0–10 mm/h). The patient also had leukocytosis with a count of 17x10^3/µl (with a normal value between 4.30 and 11.30 10^3/µL) and thrombocytosis with a count of 1547x10^3/µl (normal value between 100 and 450 10^3/µL). The Hb levels were at 9.3 g/dl (normal value is between 11.0 – 15.0 g/dl) and the HCT levels were at 28% (normal value is between 35 – 45%). However, the patient’s renal and liver profiles were normal, except for a high gamma-glutamyl transferase (GGT) enzyme level. The patient tested negative for HIV, HBV, HCV, TB, EBV and CMV.

Owing to the unusual and worrisome appearance of the skin lesions, additional diagnostic tests were performed to investigate whether the patient had any underlying systemic infection or secondary bacterial involvement. The tests included a skin biopsy for histopathological examination, blood and wound cultures, and genetic analysis.

Blood and wound cultures were collected and sent for analysis to rule out any underlying bacterial infections that may have contributed to the exacerbation of the skin lesions. These culture results were positive for Staphylococcus haemolyticus and Staphylococcus epidermidis in the blood and scant growth Escherichia coli in the wounds.

**Figure 3:**
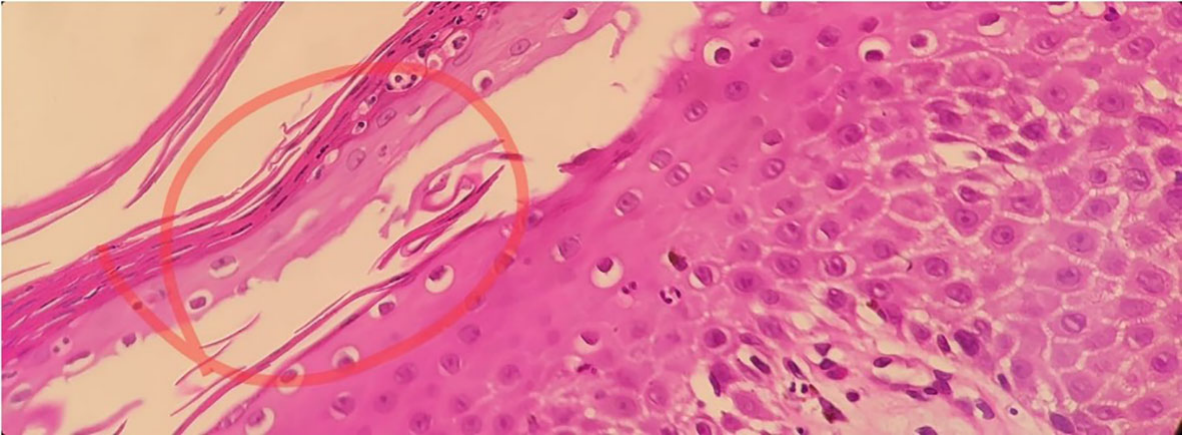
Psoriasiform hyperplasia, spongiosis (spongiform pustules of Kogoj), sub-corneal pustules, parakeratosis, diminished granular layer, thinning of the supra-papillary plate, dilated papillary dermis blood vessels with neutrophilic infiltration, superficial dermal lymphocytic infiltration, and absence of eosinophils.

Histopathological examination of a punch skin biopsy from the right leg lesions showed psoriasiform hyperplasia, spongiosis, sub-corneal pustules, parakeratosis, reduced granular layer, thinning of supra-papillary plate, dilated blood vessels in the papillary dermis with neutrophilic and monocytes infiltration, and a superficial dermal lymphocytic infiltrate (**Figure 3**). No eosinophils were detected. Considering the clinical context, these histological features strongly suggested Pustular Psoriasis.

Genetic analysis using whole-exome sequencing identified a homozygous likely pathogenic variant of IL36RN, consistent with a genetic disorder, autosomal recessive pustular psoriasis type 14 (6).

The patient was treated with Adalimumab, a subcutaneous TNF-α monoclonal antibody medication administered weekly at a dose of 20 mg, along with 1ml of oral prednisolone every other day and skin creams and ointments including: Cortiderm 1% cream, Hydrocortisone 1% cream, Liquid paraffin-white soft paraffin-glycerin cream, Daivonex 0.005% ointment, Elica 0.1% ointment, Mometasone 0.1% ointment, and White soft paraffin ointment. The lesions displayed significant regression and improvement, and nearly all had healed by the time of reporting with improvement of laboratory parameters including anemia to 12.4 g/dl, thrombocytosis to 611x10^3/µl, leukocytosis to 10.4x10^3/µl, and GGT.

## 3 Discussion

GPP is a type of psoriasis that has rarely been reported in many studies. It is also rare in children, accounting for only 0.8% of the approximately 1500 childhood psoriasis cases studied (7). Childhood GPP manifests at any age, although it is notably more frequent within the first year of life. In contrast to adulthood GPP, the childhood GPP has a higher incidence in males (8–10). Despite its rarity, early intervention is crucial, as complications can be life-threatening, including superimposed bacterial infections and sepsis (1, 11). GPP is characterized by the recurrent presence of widespread sterile pustules associated with erythema throughout the body (1, 11). The pustules can merge into a ring around the edges of the erythema and form pus-filled plaques throughout the body. Plaques tend to extend centrifugally over several hours to several days (1, 11). The other symptoms include fever and fatigue. Abnormal laboratory test results include elevated leukocytes, neutrophils, inflammatory cytokines levels, and elevated ESR (1, 11–15). Leukocytosis and elevated ESR are typically presented in GPP (13–15). Other associated abnormalities include elevated liver enzymes, increased antistreptolysin antibody, electrolyte imbalances, hypoalbuminemia, and lymphopenia (13–15). GPP is a chronic inflammatory disease that induces anemia of chronic disease by several mechanisms, such as iron sequestration in macrophages and iron-restricted erythropoiesis (16, 17). Spongiform pustules of Kogoj and Munro’s microabscesses are key features of the disease (18). Kogoj spongiform pustules are formed when neutrophils infiltrate the stratum spinosum of the epidermis. Meanwhile, Munro’s microabscesses are sites of inflammation that have a large number of infiltrating neutrophils in the stratum corneum of the epidermis (18).

Our patient exhibited erythema of the skin with a sudden appearance of erupted pus-filled lesions on the face, chest, and abdomen, along with fever and inflammation. Additionally, presented with anemia, leukocytosis, elevated ESR, and increased levels of GGT, a liver enzyme. Initially, we suspected Staphylococcal Scaled Skin Syndrome and Reter’s syndrome due to the rarity of GPP and the absence of any family history of PV and GPP. However, the patient’s unclear medical history, negative bacterial cultures, and similar presentation of his 9-month-old sibling around the same time, all indicate the possibility of an autoinflammatory disorder, which could have been triggered by an infectious factor with genetic predisposition pointing towards GPP. Therefore, skin biopsy was performed to confirm the diagnosis, and the histopathological results showed the presence of Kogoj spongiform pustules, confirming the diagnosis of GPP.

The etiology of GPP is still unknown, but several studies have indicated that it may be caused by various factors, such as infections, trauma, emotional changes, and sudden withdrawal or administration of certain medications, such as glucocorticoids and cyclosporine (12, 19). In some cases, especially in children with no history of psoriasis, the aforementioned factors may act as motivators (12). The factors underlying GPP are linked to various genes, such as IL1RN, IL36N, SERPINA1, CARD14, MPO, and AP1S3 (20). These genes are involved in the signaling pathways, particularly in the IL-1/IL-36 axis, which is the primary pathogenic inflammatory pathway that is activated in GPP (3, 21).

A study has indicated that GPP is an autoinflammatory disease caused by changes in production of interleukin-1 family proteins in the skin by disinhibiting the signaling pathway that activates these proteins (3, 22). We suspect that genetic mutations have contributed to the onset of GPP in the child in our case, considering that there was no personal or familial history of psoriasis. Genetic analysis was performed and identified the homozygous pathogenic mutation in IL36RN.

**Figure 4:**
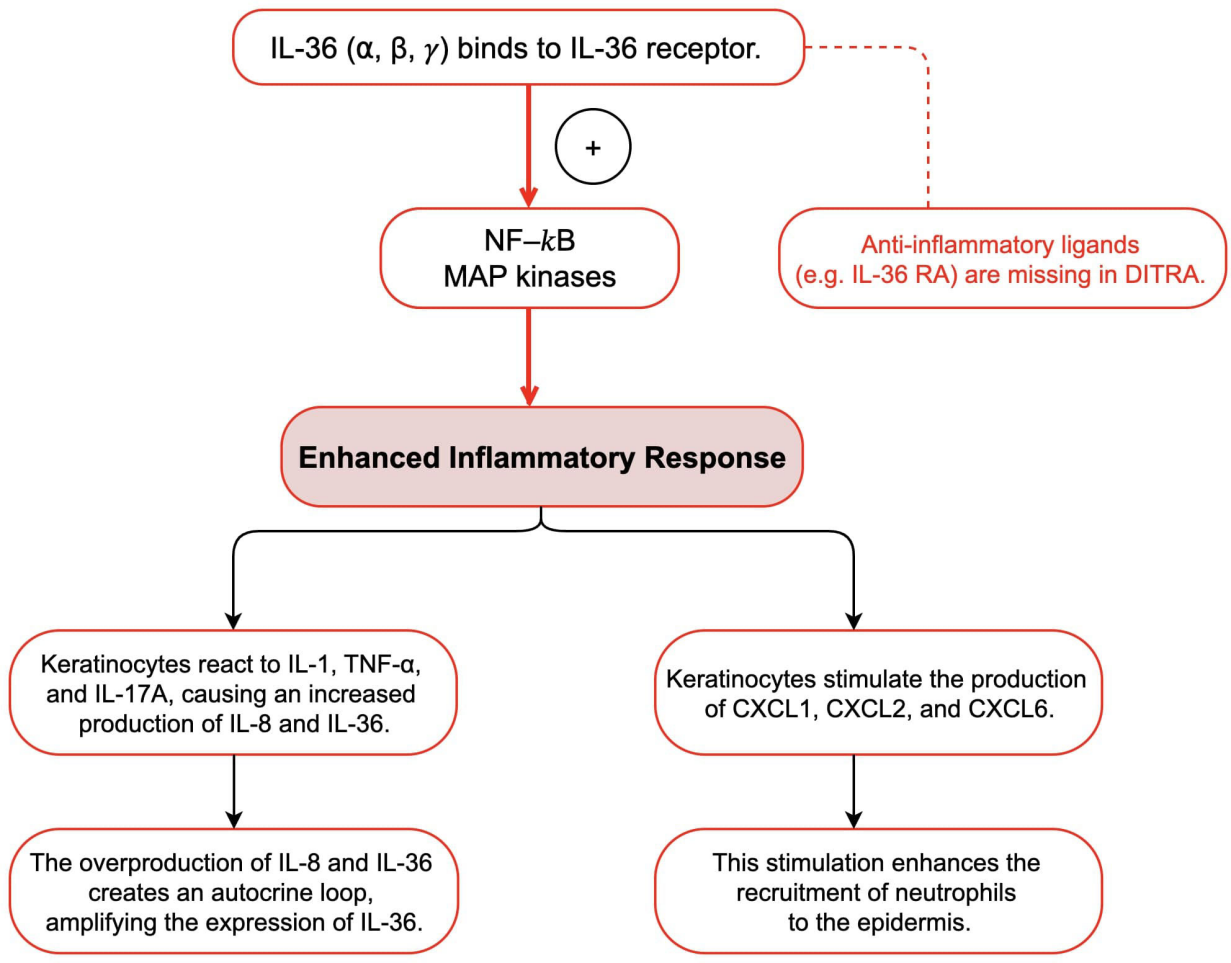
Mechanisms of IL-36Ra Deficiency and Overactive IL-36 Signaling in DITRA.

The interleukin-36 (IL-36) cytokine family includes IL-36α, IL-36β, IL-36γ, and interleukin-36 receptor antagonist (IL-36Ra) (3, 21, 22). These cytokines are expressed in multiple cell types such as epithelial tissues, keratinocytes, and immune cells (3, 21, 22). They are known to activate both nuclear factor-κB (NF-κB) and mitogen-activated protein kinase (MAPK) signaling pathways, which initiate an inflammatory response (18, 22). IL-36Ra acts as a regulator by binding to IL-36R, thereby mediating the pro-inflammatory effects of IL-36 cytokines (18, 22). IL36RN gene encodes IL-36Ra, and mutations in this gene disrupt the regulatory function of IL-36Ra, causing an overactive signaling pathway associated with IL-36, which ultimately leads to the development of GPP (3, 18, 21, 22). Mutations in the IL36RN gene that reduce IL-36Ra activity are referred to as “Deficiency of IL-36Ra” (DITRA) (22). DITRA is characterized as an independent autoinflammatory disease mediated by innate immunity (22). High fever and generalized malaise characterize DITRA during an episode (22). Moreover, several research have shown that people with a deficiency in IL36RN overproduce IL-8 and IL-36 in keratinocytes in response to various cytokines and chemokines, including IL-1, TNF-α, and IL-17A (18, 21, 22). These overproduced cytokines bind to IL-36R on the keratinocytes surface, triggering an autocrine response that further amplifies IL-36 expression (18, 21, 22). Additionally, they stimulate the production and secretion of neutrophil chemokines such as CXCL1, CXCL2, and CXCL6, which increase the recruitment of neutrophils to the epidermis (18, 22). This could explain why common infections trigger pustular flares in all patients with this condition. Several studies have reported homozygous IL36RN mutations in different families, indicating that familial GPP may follow an autosomal recessive inheritance pattern (23, 24). Furthermore, IL36RN mutations have been found in approximately one-third of the patients with GPP, including sporadic cases (12). More than 20 different mutations in IL36RN have been linked to GPP pathogenesis, with the majority occurring in Asian populations (12). Studies have shown that IL36RN mutations are more common in pediatric-onset GPP cases than in adult-onset cases, suggesting that genetic changes in IL36RN play a critical role in early onset GPP (12). This may result in a lower likelihood of plaque psoriasis and an increased susceptibility to systemic inflammation (12). These findings highlight the unique nature of GPP as a subtype of psoriasis, particularly in the presence of IL36RN mutations (**Figure 4**).

GPP causes severe systemic symptoms during an acute-phase attack, including malaise, and potentially life-threatening complications (1, 11). GPP can affect multiple organ systems, increasing the risk of sepsis, and can lead to renal and hepatic diseases, such as neutrophilic cholangitis, and respiratory abnormalities, such as neutrophilic pneumonitis and acute respiratory distress syndrome (25). Extensive pustular skin lesions can cause secondary bacterial or fungal infections, complicating the disease course and resulting in significant fluid loss and increased risk of dehydration and electrolyte imbalances (1, 11, 25). GPP can also cause psoriatic arthritis, which impairs joint mobility and leads to hepatic abscesses (25). In chronic cases, persistent systemic inflammation owing to GPP can lead to cardiovascular complications (25). The impact of GPP goes beyond physical symptoms, affecting mental well-being, and can lead to psychological stress, reduced quality of life, and even suicidal thoughts in the absence of proper support and counseling (25).

Managing childhood GPP can be challenging due to the limited research and established treatment guidelines (18, 21). Biologics, such as TNF-α inhibitors such as Adalimumab and Infliximab and IL-17 antagonists (Secukinumab), have been shown to improve prognosis and decrease complications in many cases (12). Clinical trials have investigated the effectiveness of agents targeting IL-36 or IL-36R. Their lesions rapidly improved in a trial involving GPP patients treated with a single dose of Spesolimab (an anti-IL-36R biologic) (26). While biological agents have been used, there is a scarcity of solid evidence supporting their effectiveness in treating complex cases of GPP, and the accumulation of such evidence has been slow (18, 21). Despite various case series documenting the successful use of biological agents, there is still a significant need to understand their efficacy and safety characteristics in this population (18, 21).

## Data availability statement

The datasets presented in this article are not readily available because this is a case report. Requests should be directed to the corresponding author.

## Ethics statement

The studies involving humans were approved by King Fahad Medical City (KFMC) IRB Committee (23-618). The studies were conducted in accordance with the local legislation and institutional requirements. The human samples used in this study were acquired from a by– product of routine care or industry. Written informed consent was obtained from the minor(s)’ legal guardian/next of kin for the publication of any potentially identifiable images or data included in this article.

## Author contributions

GA: Supervision, Conceptualization, Writing – review & editing, Writing – original draft. FA: Writing – review & editing, Supervision, Conceptualization. KA: Writing – original draft. ZO: Writing – original draft. EA: Writing – review & editing, Supervision. MS: Writing – review & editing, Writing – original draft.
